# Study protocol: mobile improvement of self-management ability through rural technology (mI SMART)

**DOI:** 10.1186/s40064-015-1209-y

**Published:** 2015-08-16

**Authors:** Jennifer A Mallow, Laurie A Theeke, Dustin M Long, Tara Whetsel, Elliott Theeke, Brian K Mallow

**Affiliations:** West Virginia University Health Sciences Center, Mortantown, WV USA; Sovern Run LLC, Albright, WV USA

**Keywords:** Study protocol, mHealth, eVisits, Rural, Underserved, Health disparities, Self-management, Chronic illness

## Abstract

**Background:**

There are 62 million Americans currently residing in rural areas who are more likely to have multiple chronic conditions and be economically disadvantaged, and in poor health, receive less recommended preventive services and attend fewer visits to health care providers. Recent advances in mobile healthcare (mHealth) offer a promising new approach to solving health disparities and improving chronic illness care. It is now possible and affordable to transmit health information, including values from glucometers, automated blood pressure monitors, and scales, through Bluetooth-enabled devices. Additionally, audio and video communications technologies can allow healthcare providers to conduct many parts of a physical exam remotely from varied settings. These technologies could remove geographical distance as a barrier to care and diminish the access to care issues faced by patients who live rurally. However, currently there is lack of studies that provide evidence of feasibility, acceptability, and effectiveness of mHealth initiatives on improved outcomes of care, a needed step to make the translation to implementation studies in healthcare systems. The purpose of this paper is to present the protocol for the first study of mI SMART (mobile Improvement of Self-Management Ability through Rural Technology), a new integrated mHealth intervention.

**Methods:**

Our objective is to provide evidence of feasibility and acceptability for the use of mI SMART in an underserved population and establish evidence for the refinement of mI SMART. The proposed study will take place at Milan Puskar Health Right, a free primary care clinic in the state of West Virginia. The clinic provides health care at no cost to uninsured, low income; adults aged 18–64 living in West Virginia. We will enroll 30 participants into this feasibility study with plans of implementing a longitudinal randomized, comparative effectiveness design in the future. Data collection will include tracking of barriers and facilitators to using mI SMART on patient and provider feedback surveys, tracking of patient-provider communications, self-reports from patients on quality of life, adherence, and self-management ability, and capture of health record data on chronic illness measures.

**Discussion:**

We expect that the mI SMART intervention, refined from participant and provider feedback, will be acceptable and feasible. We anticipate high patient-provider satisfaction, enhanced patient-provider communication, and improved health related quality of life, adherence to treatment, and self-management ability. In addition, we hypothesize that patients who use mI SMART will demonstrate improved physical outcomes such as blood glucose, blood pressure, and weight.

## Background

Individuals with low socioeconomic status living in rural parts of the US suffer disproportionately from poor health status, health disparities, and problems accessing healthcare. Recent data indicate that 62 million Americans current reside in rural areas, and an estimated 20 % of these individuals are underinsured. Due to state variances in implementing healthcare reform, it has been projected that the number of underinsured individuals will increase to 25 % by 2019 (DeNavas-Walt et al. 2008; Garrett et al. 2010). When compared with their urban counterparts, rural residents are more likely to (1) be economically-disadvantaged, (2) be in fair or poor health, and (3) have chronic conditions. Further, rural residents are less likely than their urban counterparts to receive recommended preventive services, and, on average, report fewer visits to healthcare providers (DeNavas-Walt et al. 2008; Garrett et al. 2010). In addition, underinsured rural adults were more likely than underinsured urban adults to report the following difficulties: poor access to care, inadequate referrals to specialists, and insufficient timeliness of care (Agency for Healthcare Research and Quality 2009).

There is a critical need for development and effectiveness testing of novel interventions that could address the social determinants of health that are responsible for health inequity (Promotion 2012). Interventions that improve access to health care and access to primary care (one of the five key domains of Healthy People 2020) have the potential to facilitate effective patient–provider communication which could thereby result in improved adherence to treatment, self-management ability, and biophysical outcome measures of common chronic conditions. The overall lack of primary care providers in rural, underserved areas further limits the implementation of healthcare system changes because additional burden may be placed on existing rural healthcare practices. Achieving improved outcomes while allowing primary care providers to deliver culturally competent and acceptable interventions that optimize time-efficiency and affordability is the real challenge (Barker et al. 2011). One potential solution could be the innovative use of mobile health (mHealth) as interventions to improve care and reduce strain on rural healthcare practices (Effken and Abbott 2009).

### Appalachia and West Virginia

Chronic illnesses such as diabetes and cardiovascular disease are more prevalent in Appalachia than other more urban regions of the United States (US) (Barker et al. 2011; Howard et al. 2011). Appalachia is a 13 state region of the Eastern US and West Virginia is in the only state that is entirely within Appalachia. West Virginia ranks 48th in the nation for lowest number of citizens with the highest underinsured population, high school graduation, highest incidence of infectious disease, highest prevalence of low birth-weight infants, and low availability of primary care providers (United Health Foundation 2012). All of these factors correspond to the five identified key domains of social determinants of health which include: economic stability, education, health and health care, neighborhood and built environment, and social and community context (Promotion 2012). The leading causes of death, illness and disability in West Virginia are chronic conditions including cardiovascular disease, diabetes, arthritis, and cancer (West Virginia Health Statistics Center). According to the Center for Disease Control, these chronic conditions are among the most prevalent, costly, and preventable of all health problems. Access to high-quality and affordable prevention measures, including screening and appropriate follow-up, are essential steps in saving lives, reducing disability and lowering costs for medical care (Sommers et al. 2012). Many healthcare system factors have been implicated in affecting outcomes, such as decreased access to healthcare, low attendance rates, and financial burden in relation to the population of interest in this study (Mallow et al. 2013; Mallow et al. 2014a, b).

### Access to healthcare in West Virginia

**Fig. 1:**
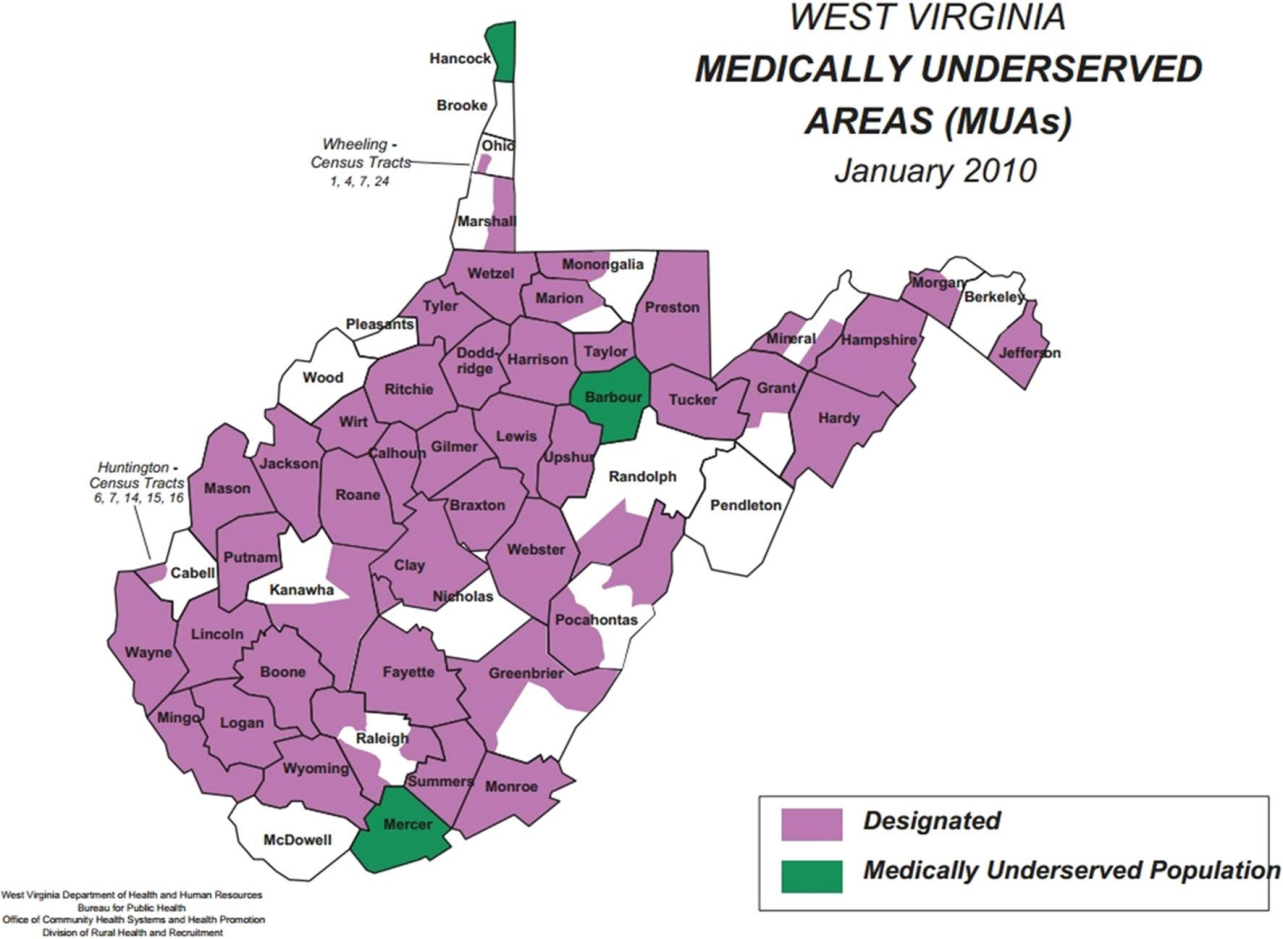
West Virginia medically underserved areas.

Of West Virginia’s 55 counties, 49 counties contain areas that are designated as medically underserved areas (purple) or medically underserved populations (green), as depicted in Fig. 1 (West Virginia Health Statistics Center 2013). Federally Qualified Health Centers (FQHC) provide care to 19.6 % of the population in West Virginia (DHHR November 2011). All persons regardless of ability to pay are able to receive care from FQHCs. The mission of FQHCs was originally meant to provide comprehensive health services to medically underserved populations to reduce the patient load on hospital emergency rooms. However, the state of West Virginia consistently ranks highest in the number of emergency room visits and preventable hospitalizations (United Health Foundation 2012). Uninsured people under age 65 averaged $1,397 in expenses for just one emergency room visit, which they paid out of pocket (Agency for Healthcare Research and Quality 2009). Additionally, even though West Virginia does have a system of free clinics, 17.7 % of the population in West Virginia reported that they could not seek medical care due to cost, which is higher than the national average of 14.6 % (Centers for Medicare and Medicaid Services 2008). Hidden costs associated with healthcare attendance include inability to quickly access care due to distance, lack of an interstate transportation system, lack of a personal automobile, lack of well-developed public transportation systems, and cost of transportation (Arcury and Gesler 2005 expert).

### Cost of chronic illness

Substantial expenditure of healthcare dollars is incurred by underinsured people with chronic illness (Yu 2009). Nationally, care for people with chronic diseases currently accounts for 83 % of healthcare spending, 81 % of hospital admissions, 76 % of all primary care visits, and 91 % of prescriptions written (Centers for Medicare and Medicaid Services 2008). For every $1 spent nationally on chronic disease prevention, $445 is spent on medical treatment for chronic diseases (Chronic Disease Directors. November 2004). It is estimated that asthma, cardiovascular disease, and chronic kidney disease alone costs West Virginia $893,778,000 annually (West Virginia Health Statistics Center 2013). West Virginia ranks among the highest in the nation in both the incidence of disease, particularly diabetes, arthritis, and cardiovascular disease, and associated risk factors such as smoking and obesity (U S Department of Health and Human Services 2012). The needs of people with chronic diseases will be the primary driver of demand for healthcare and the resulting costs for the foreseeable future.

**Fig. 2:**
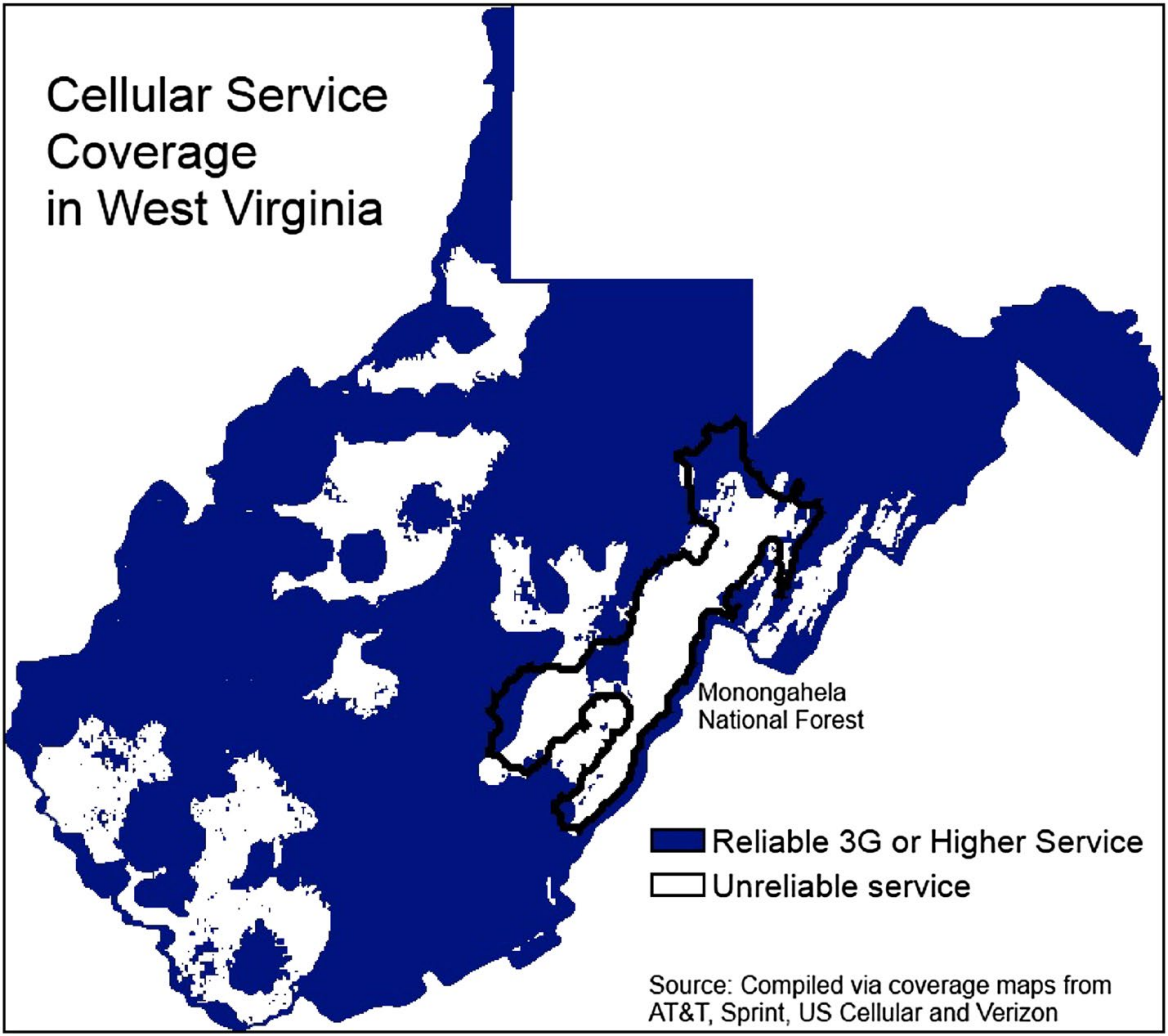
Cellular service coverage in West Virginia.

**Fig. 3:**
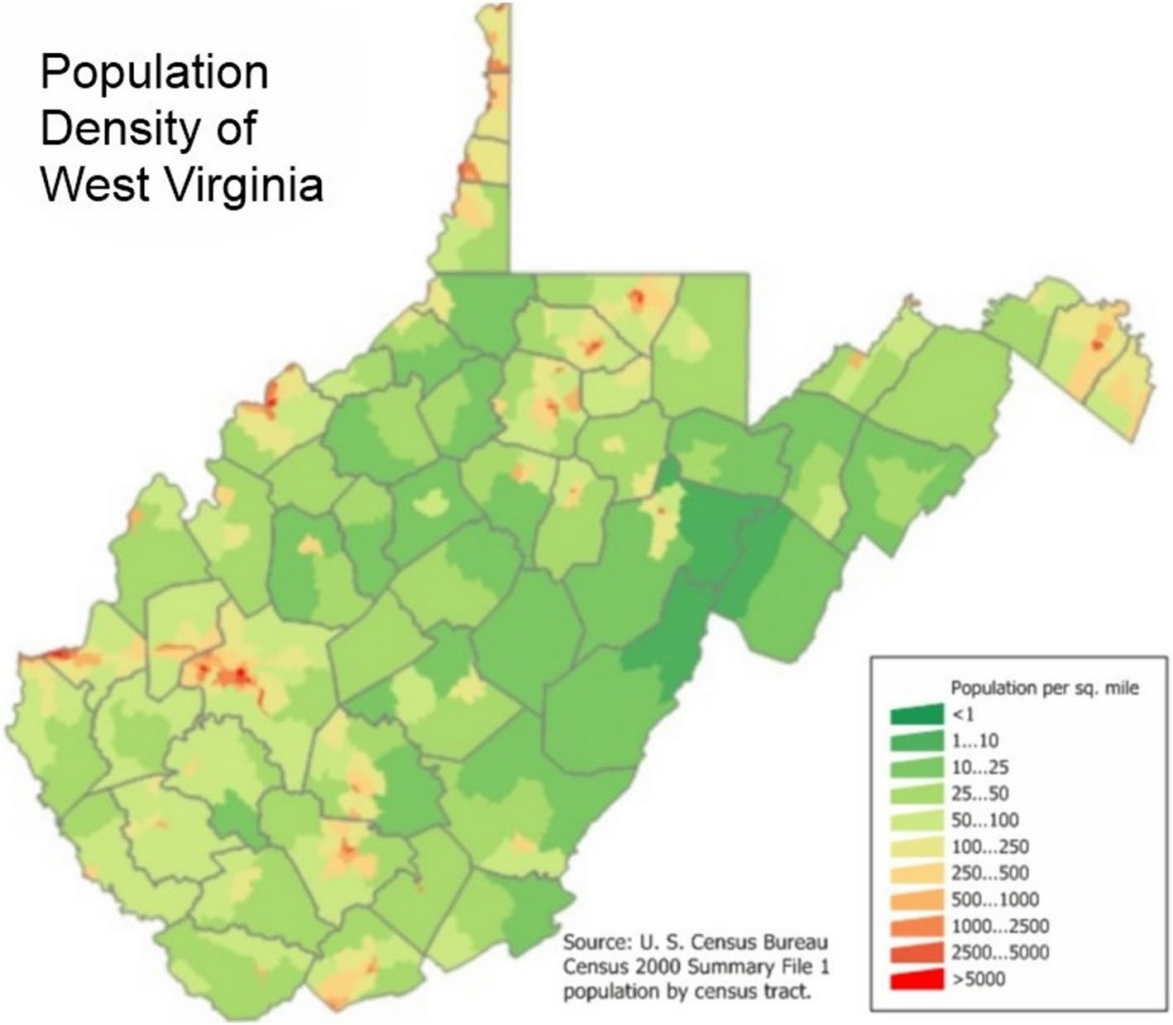
Population density of West Virginia.

*Usage of technology in Rural America* Mobile health (mHealth) is an emerging field that has been defined as “medical and public health practice supported by mobile devices, such as mobile phones, patient monitoring devices, personal digital assistants, and other wireless devices”(Cipresso et al. 2012). In the United States, there is widespread use of mobile devices and access to broadband internet service is improving (Smith 2010). Per Fig. 2, 3G service is available and reliable in the most densely populated areas of West Virginia. Much of the area where 3G service is not reliable consists of National Forest and parks (Fig. 3). Still, many of these areas have access to 1G and wired connections that could allow participation in mHealth interventions. It has been reported that even in the most rural areas of West Virginia, 77 % of adults have a cell phone (Zickuhr 2013). Currently, 88 % of American adults have a cell phone, 57 % have a laptop, and 38 % own an e-book reader or have a tablet computer. Six in ten adults (63 %) go online wirelessly with one of these devices (Zickuhr 2013). Although many technology-driven interventions has been found to improve outcomes, be cost effective, and culturally relevant (Ahern et al. 2011; Arsand et al. 2008; Ãrsand et al. 2008; Basoglu et al. 2012; Earle et al. 2010; Effken and Abbott 2009; Faridi et al. 2008; Istepanian et al. 2009; Jae-Hyoung et al. 2009; Logan et al. 2007; Lyles et al. 2011; Quinn et al. 2009; Rabin and Bock 2011; Turner et al. 2009; Yoo et al. 2009; Zolfaghari et al. 2009; Welch et al. 2015), no fully integrated systematic mHealth approach for delivery of healthcare at a distance has been studied or reported. Further, a recent systematic review of health information technology reports that continues to be a lack of evidence about the implementation and context of technology based projects (Jones et al. 2014).

### Theoretical framework for study: the chronic care model

The chronic care model has been used in the setting for this study for the past 12 years. Structuring what we know about mHealth technology using the concepts of the model adds clarity and assists with translation to clinical practice. The major concepts in the model are the health system, community support, self-management support, decision support, clinical information systems, and delivery system design (Pullicino et al. 2011). A prepared healthcare team delivering planned interactions, self-management support with effective use of community resources, integrated decision support, and supportive information technology (IT) are designed to work together to strengthen the provider–patient relationship and improve health outcomes (Pullicino et al. 2011). The will focus on self-management support, clinical information systems, and delivery system design, health system, decision support, and community support.

While previous approaches were effective in improving outcomes, they have not resulted in the establishment of the necessary electronic infrastructure for a sustainable mobile healthcare delivery model. A delivery system redesign was needed to develop a patient-centered clinical information systems within the rural health care clinic setting. The project has one aim with two sub-aims:

### Specific aim 1: establish an mHealth technology-based healthcare delivery model for use in an underserved, rural population

- *Sub aim 1a: Develop a healthcare delivery model that includes mHealth technologies* The approach will involve establishment of the necessary electronic infrastructure for mobile healthcare delivery to facilitate timely and effective healthcare at a distance. We call the delivery platform mI SMART (mobile improvement of self-management ability through rural technology). (pronounced MY SMART).
- *Sub aim 1b: Evaluate the feasibility and acceptability of mHealth care delivery in an underserved population* The approach will involve the identification of at-risk patients to pilot test this empirically-grounded strategy and measure feasibility, acceptability, and outcomes of care, including: patient–provider communication, adherence to treatment, self-management ability, quality of life, blood glucose, blood pressure, and weight.

## Methods/design

### Sub aim 1a

In order to improve rural health disparities and access to affordable health care, development of a healthcare delivery model that includes mHealth technologies is needed. The objective for Sub aim 1a is to facilitate effective patient-provider communication by removing the barrier of distance through the use of technology. The necessary electronic infrastructure for mobile healthcare delivery requires: development of a mobile application for the patients to access the mobile healthcare clinic, development of a HIPAA (Health Insurance Portability and Accountability Act) compliant virtual audiovisual visit, development of the web based application for healthcare providers to access patients and patient records, creation of a web service for the communication between the mobile devices and a record database, and the integration of the record database into the existing Electronic Medical Record (EMR). This entire platform is called mI SMART. The rationale for this aim is that successful completion of the proposed project will contribute a missing, fundamental element to our ability to provide rural healthcare, without which the potential of reversing the growing number of people in poor health with chronic illnesses in rural settings in the US will remain limited. When the proposed development of mI SMART has been complete, it is our expectation that mHealth and distance visit technologies will have been integrated into existing rural health clinics, allowing for the first time the implementation of a novel and much-needed approach to rural healthcare delivery.

### Security practices

All data that will be transmitted across the Internet will be encrypted so that patients’ private health information will be protected. Additionally, all patients and healthcare providers will receive a unique username and password to access the system.

### Development of a mobile application for patients to access the mobile healthcare clinic

Each participant will be provided a Nexus 7 tablet and Bluetooth enabled glucometer, blood pressure cuff, and scale. The application that the patient will be able to access via their mobile device, the Nexus 7 (3G tablet), is being developed using Visual Studio, an Integrated Development Environment (IDE), using the programming language Visual Basic.Net. This study will use the Bluetooth enabled: glucometer, blood pressure cuff, and scale. However, the application developed will be able to expand to other health monitoring devices in the future. The application will be able to receive the information from the mobile health monitoring devices and upload that information using a web service into a database. Through this application, the patient will also be able to securely message healthcare providers, attend live educational sessions and healthcare visits through HIPAA compliant video teleconferencing, and receive timely feedback on glucose and blood pressure readings.

### Development of the web-based application for healthcare providers to access patients and patient records

This web-based application will be developed in Visual Studio, an IDE, using programming language Visual Basic.NET. This web application will be hosted on a Windows 2012 server to be located in a secure West Virginia University data center. This application will allow healthcare practitioners to access the uploaded data from the mobile health monitoring devices and provide feedback on readings through a web service into a database. Critical clinic values, such as dangerously high blood pressures or blood glucose readings, will be flagged in the system and immediately used to notify the Registered Nurse who will refer as needed. Through this application, the healthcare practitioners will also be able to securely message patients, and deliver live educational sessions and healthcare visits through HIPAA compliant video teleconferencing.

### Creation of a web service for the communication between the mobile devices and a record database

A Web service is a method of communication between two electronic devices via the Internet. This is needed so that the Bluetooth devices and the Web-based application can communicate with the database. The web service provides data security. The database will not be in direct communication with the Internet and can only be accessed securely via the web service, thereby protecting patients’ private health information.

### Testing the mobile and web-based application

At the completion of the development and prior to testing the intervention with patients, members of the research study team will be asked to participate in testing the application. We will assess the reliability of the mobile health devices to send information to the database and general messaging and feedback components of the application. We will ask our members to take daily finger-sticks, blood pressures and weights and transmit the information to the database. The local memory of the mobile devices will be compared to the information received by the database. Qualitative data will be collected regarding usability of the application. Revisions will be made if necessary prior to intervention delivery with patients.

### Integration of the record database into the existing electronic medical record (EMR)

After the development and implementation of the intervention, we will develop a method of integrating our mHealth data into the EMR. This will be developed in Visual Studio, an IDE, using programming language Visual Basic.NET. This will be accomplished by a Windows 2012 service that will synchronize data from the database into the EMR on an hourly basis. Doing this will allow seamless integration of patient health information to all healthcare providers in the clinic. Integration and documentation of care into the EMR is the next logical step in the continuum of developing an integrated rural mHealth delivery model.

### Expected outcomes of Sub aim 1a

At the completion of this process, we expect to have created a platform, mI SMART, that will allow patients to access the mobile healthcare clinic, providers to access patients and patient self-monitoring records across geographical distances, a secure web service that provides secure communication between mobile devices and a record database, and the integration of that record database into the existing Electronic Medical Record (EMR). Hence, development of mI SMART is expected to have enabled us to implement and evaluate a novel and much-needed approach to rural primary-care healthcare delivery.

### Sub aim 1b

To improve overall health status and the ability of patients to attend scheduled clinic visits in rural settings, a new approach to solving rural health disparities is needed. The objective for this Sub aim 1b is to evaluate the feasibility, acceptability, and outcomes of care using mI SMART. To attain the objective for this aim, we will test the feasibility and acceptability of mHealth care delivery through identification of at-risk patients and measure outcomes of care, including patient–provider communication, adherence to treatment, self-management ability, quality of life, blood glucose, blood pressure, and weight. We will give at participants an Android tablet, a Bluetooth enabled glucometer, blood pressure cuff, and scale along with a training session on how to use the devices. The replacement of broken or damaged equipment will be managed by the project coordinator and sent to participants via postal mail or patient pick-up at the clinic. We will pursue an approach of combined activity logging and questionnaires to evaluate provider communication, adherence to treatment, feasibility, and acceptability. We will use a pre/post design with no randomization or control group. Because of budgetary constraints, we will enroll 30 participants into the study. A larger randomized control trial is planned after analysis of the results of this study.

### Setting

The proposed study will take place at Milan Puskar Health Right, a primary care clinic that provides health care at no cost to uninsured, low income, adults aged 18–64 living in West Virginia. The clinic provides direct healthcare, medications, and health education for this patient population. The clinic has strong ties to West Virginia University Health Science Center and many of the healthcare providers at the clinic are faculty or former students.

### The intervention

The creation of mI SMART is being guided by the model for developing complex nursing interventions (Corry et al. 2013). The multi-dimension technology intervention is designed to replicate what is accomplished through standard in-person outpatient primary care visits. The distance intervention will last for 12 weeks. Twelve weeks were chosen so that a change in biophysical outcomes could be detected and so that preliminary data could be analyzed in order to begin developing the subsequent application toward the end of the 2nd year of this grant. The participants will receive education related to self-management of their chronic illness via the developed video teleconferencing system. The curriculum for the education was developed by JM and TW and adapted from the National Standards for Diabetes Self-Management Education and Support (Haas et al. 2013) and will be delivered by a team consisting of a Nurse Practitioner, a Pharmacist (PharmD) or a Board Certified in Advanced Diabetes Management professional. The curriculum has been tested in previous interventions in this clinic and found to be effective. Using the developed video teleconferencing system, the patients will be provided tailored education about blood glucose monitoring, medication, nutrition, exercise, foot care, heart disease, complications of chronic illnesses, and behavior change. A Nurse Practitioner will perform a limited physical exam, a through health history, medication adjustments, appropriate referrals and a cardiovascular risk factor assessment with treatment as indicated for all abnormal results via the developed video teleconferencing system. A Registered Nurse will review and provide feedback and appropriate referral for finger stick logs, blood pressure logs and weights via the developed secure messaging system.

### Identification of at-risk patients

At-risk patients are those patients for whom distance to the clinic is greater than the average distance and who are diagnosed with a chronic illness including diabetes, obesity, hypertension, or hyperlipidemia. This was chosen so that feasibility and acceptability could be assessed in this difficult population and so that one of the monitoring devices used will monitor one of the diagnosed chronic illnesses. Our previous pilot studies using the EMR of the rural healthcare clinic where the intervention will take place have identified that mean travel distance to this clinic for patients is 21 miles. The clinic has more than 28,000 patient encounters annually and provided chronic illness care to 1,734 patients in 2013. Of the patients with chronic illnesses, 637 patients live greater than 21 miles from the clinic. Given these parameters, the identification of 30 participants is feasible and will be done through the recommendations of Nurse Practitioners in the clinic and inclusion criteria.

### Potential participants

Inclusion criteria include being an adult age 18–64 with a diagnosis of a chronic illness and receiving care at the free clinic. The free clinic where the trial takes place does not accept patients with Medicaid. Hence, those over the age of 65 do not attend this particular clinic. Study participants will be of both genders. Exclusion will include participants who do not speak or read English at a 3rd grade reading level, and those with dementia or psychosis that would prevent on-going education and communication.

### Demographics

Demographics will be collected so that descriptive reports of the sample can be reported. The following demographics will be collected: Age, Gender, Ethnicity, Marital Status, Education, Income, Employment status, co-morbidities, duration of chronic illness, number of people in household, distance from clinic.

### Feasibility and acceptability

The use of the developed technology to deliver the intervention and to collect and store data will be evaluated in four ways. We will assess the ability of participants to use the developed technology to receive and transmit health information by: (1) reviewing the presence or absence of data in the database from each participant, (2) assessing the electronic activity logs and error messages for each participant, (3) assessing the electronic activity logs and error messages for each provider (4) analyzing the electronic activity logs of each application for number of times each application was used as well as common errors. Lastly, we will assess the acceptability of the technology with post intervention electronic patient and provider satisfaction questionnaires.

### Patient–provider communication and satisfaction

Participants will be provided questionnaires to assess communication based on 5-point Likert scale. We will evaluate ease and convenience of communication, promptness of replies, quality and amount of information, and quality of care. In addition, participants will assess their satisfaction with the overall system. The provider’s communication and satisfaction will measure the amount of time to review electronic logs and reply to electronic messages, their promptness in responding to messages, volume of messages, convenience of the system, quality of the information, and satisfaction with the electronic communication. All communication requiring interaction between the patient and health care provider will be stored in an activity log. The activity log will be analyzed for frequencies of all patient-provider communication.

### Adherence to treatment

The number of blood glucose, blood pressure and weight measurements uploaded to the database will be compared to the number of provider-requested measurements. Additionally, it is expected that trending improvement in blood glucose, blood pressure, and weight will be seen.

### Self-management ability

We will measure self-management ability with the American Association of Diabetes Educators (AADE) Behavior Score Dashboard (BSD). This tool is free to use for practicing diabetes educators and is already used by the clinic. It is designed to use with patients to examine all areas of self-management. The assessment includes 21 questions related to behaviors such as healthy eating, being active, monitoring, and taking medications. This tool will be used to provide a patient specific framework for individualized diabetes education. Additionally, we will use the tool as a means of measuring both participant progress and mI SMART outcomes. This tool is recommended by the AADE. Face validity of the instrument was established by an expert panel put together by the AADE. The expert panel found the BSD to be consistent across questions, clear and readable at lower literacy levels. The expert panel also determined that the questions were well constructed and understandable (Tomky 2011). However, psychometric measurements of validity and reliability have not yet been published. Participants will take this survey electronically prior to intervention and after 12 weeks of care.

### Quality of life and functional ability

Quality of life, loneliness, and depression will be measured to assess the base line patient perceived quality of life, loneliness and depression. Post intervention measures will be used to assess the differences in these measures after this intensive support intervention.

*Quality of life* The Medical Outcomes Trust Short Form-36 Health Survey versions 2.0 (SF-36v2), will be used to will be measure quality of life. The SF-36v2 is a 36-item questionnaire that reflects eight general health concepts including physical functioning (10-item), role-physical functioning (4-item), bodily pain (2-item), mental health (5-item), role-emotional functioning (3-item), social functioning (2-item), vitality (4-item), and general health (5-item). Each item is coded with a numerical value, summed, and transformed to a scale ranged from 0 to 100 (the higher score, the better state of health). Reliability and validity of the SF-36 is supported by many studies. The instrument is easy to administer in 5–10 min. Participants will take this survey prior to intervention and after 12 weeks of care (McHorney et al. 1993). *Loneliness* The 20 item University of California, Los Angeles (UCLA) Loneliness Scale (version 3) will be used to assess loneliness. It reflects a conceptualization of loneliness as a complex phenomenon with both emotional and social components. The current version, version 3, appeared in 1996 and includes 11 positively worded and nine negatively worded items. All items can be answered using a Likert scale, with potential answers of “never,” “rarely,” “sometimes,” and “often”; each answer is assigned a point value ranging from 1 (never) to 4 (often). Possible total scores range from 20 to 80, with 20 indicating no loneliness and higher scores indicating greater loneliness. Scores over 40 are generally considered to indicate loneliness. The scale has high internal consistency (a Cronbach α of 0.89–0.94)and positive test retest reliability (r = 0.73) (Russell and Cutrona 1980). Participants will take this survey prior to intervention and after 12 weeks of care.

*Depression* The Patient Health Questionnaire (PHQ-9) is a 9 item multipurpose instrument for screening, diagnosing, monitoring, and measuring the severity of depression. The tool rates the frequency of depressive symptoms as well as the presence and duration of suicidal ideation. The PHQ-9 can be completed in a few minutes and can be administered repeatedly to assess for improvement or worsening of depression. The tool had a sensitivity of 88 % and a specificity of 88 % for major depression. Scores of 5, 10, 15, and 20 represent mild moderate, moderately sever and severe depression (Kroenke et al. 2001). Participants will take this survey prior to intervention and after 12 weeks of care.

*Blood glucose, blood pressure, and weight* The physical measures of body weight, blood glucose and blood pressure will be measured because these are commonly collected at clinic visits as measures of chronic illness control. Participants will have body weight, blood pressure, and blood glucose obtained upon enrollment into the study by the blue-tooth enabled scales, blood pressure monitors, and glucometers. These Bluetooth-enabled devices will send the readings directly to the study database. Participants will continue to perform these physical measures and have these measures recorded at home with provided equipment as often as directed by their primary care provider. The mI SMART system will also provide automated prompts to obtain readings along with automated immediate feedback for readings within normal limits and timely feedback from study nurses with appropriate referral for abnormal readings. Goals for self-monitoring are set by the primary care provider, are patient specific, and are displayed as green for normal results, yellow for slightly low or high readings and red for critical values.

### Analysis of data

Data analyses will be facilitated by Dr. Dustin Long, the bio-statistician on our team, using SAS (Cary, NC) for Windows, version 9.3. For this feasibility and acceptability trial, we will be using a pre/post design. Techniques for assessing differences before and after the mI SMART intervention will depend on the type of outcome. For outcomes which are scaled, i.e., patient-provider satisfaction, the quasi-likelihood analysis will be used to determine if the intervention had an effect on the distribution of scores. For continuous outcomes, i.e., blood glucose, blood pressure, weight, the standard *t* test will be used to test differences in means before and after intervention. If the data are not normally distributed, the non-parametric Wilcoxon rank sum test will be used. For count measures, i.e., patient-provider communication, Poisson regression will be performed to assess the difference in mean counts before and after intervention. For measures that are percentages, i.e., for adherence to treatment measures (as patients will only measure themselves a certain percentage of the prescribed number) logistic regression will be performed to determine the difference pre and post intervention. Each of these analyses will be performed at a significance level of 0.05.

### Expected outcomes of Sub aim 1b

The rationale for this aim is that successful completion of the proposed research will contribute a missing, fundamental element to our base of knowledge, without which the feasibility and acceptability of mHealth care delivery in an underserved, rural population will be unknown. The acquisition of such knowledge is critical to the development of improved interventions for at risk rural patients with chronic illnesses. When the proposed studies for sub aim 1b have been completed, it is our expectation that mI SMART will have been found to be feasible and acceptable to rural patients and rural healthcare providers and that overall outcomes of care will have been improved. Such a finding would be of importance because it would constitute a crucial step forward in diminishing health disparities and improving overall health status in underserved populations.

## Discussion

There is an absence of the necessary electronic infrastructure needed to implement mobile healthcare delivery in rural underserved areas. Findings of a recent literature review we conducted indicated that individual interventions using mobile technology can positively impact outcomes of chronic illness while at the same time reduce the cost and burden to patients However, no approach to date has combined the individual interventions as an integrated system to deliver healthcare at a distance within existing rural health clinics. The ability of such interventions to improve care and reduce strain on rural healthcare practices will depend on the effective use of technology (Effken and Abbott 2009). Our team of experienced rural healthcare clinicians, researchers, a technology developer, project manager, and a statistician is uniquely suited to develop, implement, and evaluate a healthcare delivery model that includes mHealth technologies in a rural population.

The team has completed several intervention studies in addition to two preliminary studies at the free clinic proposed for use in this study. The first study at this clinic was an intervention study that involved testing the effectiveness of Group Medical Visits (GMVs) with a sample of 111 patients with a chronic illness receiving care at the rural free clinic. The intervention, intended to cluster care, included group education and elements of an individual patient visit. There were 53 participants who attended GMVs and 58 participants who received usual care. The majority of patients were female, white, morbidly obese, had a high-school education or less, were age 50 or younger, had a mean of 5 co-morbid conditions, and drove long distances to receive care. At baseline, the patients who attended GMVs had higher A1C levels, reported more pain, had increased depression levels and were more obese than those who received usual care. There was a statistically significant decrease in systolic blood pressure from time one to time two in patients who attended GMVs. There was no significant impact on outcomes of patients who received usual care from time one to time two. However, it is important to note that the majority of patients attended two or fewer visits in 1 year. Previous studies reviewed related to GMVs suggest that improved interventions are seen in those patients who attend more frequently (Trento et al. 2001, 2002; Beck et al. 1997). Hence, the lack of improvement in biophysical outcomes of care in this sample of patients who attended GMVs may be due to low attendance rates. The limited impact of this traditional style intervention in relation to low attendance argues for the need to test alternative interventions to reach this difficult population.

As a follow up, we conducted a study that involved the development of a computer program to extract data from the Electronic Medical Record, clinic scheduling system, and free clinic pharmacy records into a de-identified analyzable data source to investigate the relationship between attendance at clinic, patient characteristics, and biophysical outcomes of low-income, uninsured persons with chronic illnesses. The study sample consisted of 6,314 patients who received care for a chronic illness at the free clinic from May 2008 through May 2012. Patient characteristics collected include: Age, gender, ethnicity, marital status, duration of chronic illness, education, distance from clinic, co-morbidities, number of medications, type of medications and depression. Common outcomes of chronic illness care were collected including: A1C, weight, blood pressure, fasting glucose, lipids, hypoglycemic episodes, depression, end organ damage, and microalbumin. Results indicate that 42 % of the scheduled patients did not attend their free healthcare visits. Patients who lived farther than 30 miles from the clinic and had more than one chronic illness were more likely to miss scheduled follow-up care. Additionally, the patients who missed their schedule follow-up care had higher A1C, blood pressure and lipid levels and higher reports of depression than those who did not miss their scheduled follow up visits (Kroenke et al. 2001).

## Conclusion

The status quo with respect to rural healthcare places the burden of caring for chronic illness on patients who have very few resources. The cost of travel due to long distances between rural healthcare clinics and patients’ homes frequently prevents patients from seeking needed healthcare (Arcury et al. 2005). Results of our studies highlight that patients who live longer distances from the rural clinic are more likely to miss scheduled chronic illness follow-up appointments. This evidence strongly suggests that removing distance through the use of technology to overcome the issues rural patients face regarding access to care is a promising approach to improve timeliness of care. The research proposed in this protocol is innovative, in our opinion, because it represents a new and substantive departure from the status quo, namely the approach of integrating multiple mHealth tools into an existing rural health clinic to go beyond traditional office visits and shifting to real-time exchanges between patients and providers across geographical boundaries. As a consequence, an efficacious shift in the traditional rural healthcare delivery paradigm to one that uses technology to improve outcomes of care is expected to result.

Although there is little ethnic diversity in West Virginia, we have a unique opportunity to study a vulnerable and understudied group. Because samples from most clinical studies are recruited from healthcare providers at medical centers, a notably under-studied group has been those who are uninsured or underinsured and receive low cost and free care. Yet, it is precisely this group, who usually has multiple chronic illness and many barriers to self-management that is of interest to researchers and healthcare providers as they attempt to reduce health disparities. By collaborating with Health Right, we have an opportunity to enroll these individuals and thus include a heretofore “hard-to-reach” population. By using community healthcare providers and based on our previous work, we anticipate high rates of identification, recruitment and retention of this underserved population living in a highly distressed environment. In addition, our experience suggests that we will enroll roughly equal numbers of women and men, and will have no trouble including women, thus, providing us with the opportunity for meaningful, comparisons between men and women. We also will enroll adults between the ages of 18–64, an understudied group when looking at chronic illness.

## Abbreviations

AADE: American Association of Diabetes Educators
BSD: behavior score dashboard
EMR: electronic medical record
FQHC: federally qualified health centers
GMVs: group medical visits
HIPAA: health insurance portability and accountability act
IT: information technology
IDE: integrated development environment
mHealth: mobile health
mI SMART: mobile improvement of self-management ability through rural technology (pronounced my smart)
PHQ-9: patient health questionnaire
SF-36v2: medical outcomes trust short form-36 health survey versions 2.0
UCLA: University of California, Los Angeles
